# Functional characterization of a gamma-glutamyl phosphate reductase ProA in proline biosynthesis and promoting expression of type three secretion system in *Ralstonia solanacearum*

**DOI:** 10.3389/fmicb.2022.945831

**Published:** 2022-08-29

**Authors:** Yue Guan, Rongsheng Wang, Nan Chen, Yalan Zhu, Liangliang Han, Xinping Chen, Jing Li, Yong Zhang

**Affiliations:** ^1^College of Resources and Environment, Southwest University, Chongqing, China; ^2^Liaoning Key Laboratory of Plant Pathology, College of Plant Protection, Shenyang Agricultural University, Shenyang, China; ^3^College of Chemistry and Chemical Engineering, Chongqing University of Science and Technology, Chongqing, China; ^4^Interdisciplinary Research Center for Agriculture Green Development in Yangtze River Basin, Southwest University, Chongqing, China; ^5^The Ninth People’s Hospital of Chongqing, Chongqing, China

**Keywords:** *Ralstonia solanacearum*, type three secretion system (T3SS), pathogenesis, ProA, amino acid biosynthesis

## Abstract

*Ralstonia solanacearum* RSc2741 has been predicted as a gamma-glutamyl phosphate reductase ProA catalyzing the second reaction of proline formation from glutamate. Here, we experimentally demonstrated that *proA* mutants were proline auxotrophs that failed to grow in a minimal medium, and supplementary proline, but not glutamate, fully restored the diminished growth, confirming that ProA is responsible for the biosynthesis of proline from glutamate in *R. solanacearum*. ProA was previously identified as one of the candidates regulating the expression of genes for type three secretion system (T3SS), one of the essential pathogenicity determinants of *R. solanacearum*. Supplementary proline significantly enhanced the T3SS expression both *in vitro* and *in planta*, indicating that proline is a novel inducer of the T3SS expression. Deletion of *proA* substantially impaired the T3SS expression both *in vitro* and *in planta* even under proline-supplemented conditions, indicating that ProA plays additional roles apart from proline biosynthesis in promoting the expression of the T3SS genes. It was further revealed that the involvement of ProA in the T3SS expression was mediated through the pathway of PrhG-HrpB. Both the *proA* mutants and the wild-type strain grew in the intercellular spaces of tobacco leaves, while their ability to invade and colonize tobacco xylem vessels was substantially impaired, which was about a 1-day delay for *proA* mutants to successfully invade xylem vessels and was about one order of magnitude less than the wild-type strain to proliferate to the maximum densities in xylem vessels. It thus resulted in substantially impaired virulence of *proA* mutants toward host tobacco plants. The impaired abilities of *proA* mutants to invade and colonize xylem vessels were not due to possible proline insufficiency in the rhizosphere soil or inside the plants. All taken together, these results extend novel insights into the understanding of the biological function of ProA and sophisticated regulation of the T3SS and pathogenicity in *R. solanacearum*.

## Introduction

The syringe-like type three secretion system (T3SS) is essential for the pathogenicity of many pathogenic bacteria in animals and plants, which enables productive interaction between bacteria and eukaryotic host cells by delivering virulence proteins (called type III effectors, T3Es) directly into the cytosol of host cells to usurp and subvert host defense (Cornelis, 2006; Galán and Wolf-Watz, 2006). *Ralstonia solanacearum* is a causal agent of bacterial wilt disease in 450 plant species worldwide, and the T3SS plays an essential role in the infection process of *R. solanacearum*, particularly at the early stage of invading host cells (Cunnac et al., 2004; Angot et al., 2006; Jones and Dangl, 2006). *R. solanacearum* is extremely heterogeneous and currently regarded as a *Ralstonia solanacearum* species complex (RSSC), including *R. solanacearum* and closely related species of *R. syzygii*, *R. picketti*, and banana blood disease (BDB) bacterium. The T3SS is greatly conserved among the RSSC, which is encoded by 22 genes arranged in a regulon, named the hypersensitive response and pathogenicity (*hrp*) regulon, and is responsible for a hypersensitive response in resistant plants and pathogenicity in host plants (Arlat et al., 1992; Genin and Denny, 2012; Coll and Valls, 2013).

To date, the *R. solanacearum* T3SS has been well demonstrated to be globally regulated by a complex network (Valls et al., 2006; Genin, 2010; Hikichi et al., 2017). In brief, the expression of the T3SS and entire T3Es genes (more than 100 T3Es in the RSSC) was directly controlled by a key regulator HrpB, an AraC-family of transcriptional regulator, which binds directly to the *hrp*_*II*_ motif in the promoters of many target genes to control their transcription (Cunnac et al., 2004; Mukaihara et al., 2004, 2010). The expression of *hrpB* and the entire T3SS genes is not activated in a nutrient-rich medium until the bacterium gets in contact with host signals or some mimic signals, such as those in a nutrient-limited medium that might mimic plant apoplastic fluids to activate the T3SS expression, so-called a *hrp*-inducing medium (Marenda et al., 1998; Angot et al., 2006; Yoshimochi et al., 2009b). Two close paralogs HrpG and PrhG, which are OmpR/PhoB family of response regulators of the two-component system (TCS), respond to host signals or mimic signals by phosphorylation and positively regulate *hrpB* expression in parallel ways (Plener et al., 2010; Zhang et al., 2013). Host signals or mimic signals are presumed to be recognized by the outer membrane protein PrhA and transferred to HrpG via a signaling cascade of PrhA-PrhR/PrhI-PrhJ or some unknown cascades (Genin and Denny, 2012; Hikichi et al., 2017). Although both HrpG and PrhG positively regulate *hrpB* expression in parallel ways, HrpG is essential for host invasion, host colonization, and pathogenicity, while PrhG contributes partially to pathogenicity and host colonization. HrpG is presumed to respond to host signals, while PrhG seems to respond to some metabolic signals (Plener et al., 2010; Zhang et al., 2013; Pedro-Jove et al., 2021). In addition, a global virulence regulator PhcA, which is a LysR family of the transcriptional regulators and quorum sensing-dependent, negatively regulates *hrpG* expression, but positively regulates *prhG* expression (Genin et al., 2005; Yoshimochi et al., 2009a; Zhang et al., 2013). *R. solanacearum* might switch from using HrpG to PrhG for *hrpB* activation in a cell density-dependent manner (Zhang et al., 2013).

To further elucidate the complex regulation of the T3SS in *R. solanacearum*, we previously isolated some transposon mutants, in which the T3SS expression was substantially decreased in *R. solanacearum* strain OE1-1, a pathogenic strain of tomato and tobacco plants (Kanda et al., 2003; Zhang et al., 2013). The expression profiles of the T3SS were monitored with a *popA-lacZYA* fusion, which exists as part of a *popABC* operon with *popB* and *popC*, and is mapped to the left side of the *hrp* regulon (Salanoubat et al., 2002). PopABC includes three T3Es, which are currently renamed as RipS (*Ralstonia*-injected proteins) of RipX, RipAB, and RipAC, respectively, and the expression of *popABC* is directly controlled by the master regulator HrpB (Peeters et al., 2013; Landry et al., 2020). The expression of *popA-lacZYA* exhibits similar profiles to the T3SS genes under different conditions, and this construction does not alter the infection process of OE1-1 toward different host plants (Yoshimochi et al., 2009b; Zhang et al., 2011; Chen et al., 2022). Among them is RSc2741 (426 amino acids in GMI1000, a well-studied *R. solanacearum* strain), which has been predicted as a gamma-glutamyl phosphate reductase ProA catalyzing the second reaction of proline formation from glutamate (Salanoubat et al., 2002; Hoffmann et al., 2017). The expression level of *popA-lacZYA* (the T3SS) was substantially decreased in the *proA* transposon mutants, indicating ProA possibly exerts a novel effect on the expression of the T3SS genes. This is the first report linking the T3SS with proline biosynthesis. We thus focused on ProA to investigate its contribution to the T3SS and pathogenicity in *R. solanacearum*.

## Materials and methods

### Bacterial strains and culture conditions

*Ralstonia solanacearum* strains used in this study were derivatives of *R. solanacearum* OE1-1 (Table 1), which is virulent in tomato and tobacco plants (Kanda et al., 2003). *E. coli* strains of DH12S and S17-1 were grown at 37°C in LB medium, which were used for plasmid construction and conjugational transfer, respectively. *R. solanacearum* strains were grown at 28°C in a nutrient-rich medium (broth medium) and a minimal medium (Hoagland medium with 2% of sucrose, used as a *hrp-*inducing medium) (Yoshimochi et al., 2009b).

**TABLE 1:** Bacterial strains used in this study.

Strain	Relative characteristics	References
OE1-1	Wild-type, race 1, biovar 3	Kanda et al. (2003)
RK5043	OE1-1, *phcA-lacZYA*	Yoshimochi et al. (2009b)
RK5046	OE1-1, *hrpB-lacZYA*	Yoshimochi et al. (2009b)
RK5050	OE1-1, *popA-lacZYA*	Yoshimochi et al. (2009b)
RK5120	OE1-1, *hrpG-lacZYA*	Yoshimochi et al. (2009b)
RK5124	OE1-1, *prhJ-lacZYA*	Yoshimochi et al. (2009b)
RK5212	OE1-1, *prhG-lacZYA*	Zhang et al. (2013)
RK5619	OE1-1, *prhN-lacZYA*	Zhang et al. (2015)
RK5130	OE1-1, *prhIR-lacZYA*	Yoshimochi et al. (2009b)
RQ5735	*popA-lacZYA*, Δ*proA*	This study
RQ5935	*hrpB-lacZYA*, Δ*proA*	This study
RQ5966	*hrpG-lacZYA*, Δ*proA*	This study
RQ6043	*prhG-lacZYA*, Δ*proA*	This study
RQ6074	*prhJ-lacZYA*, Δ*proA*	This study
RQ6138	*prhN-lacZYA*, Δ*proA*	This study
RQ6141	*phcA-lacZYA*, Δ*proA*	This study
RQ6144	*prhIR-lacZYA*, Δ*proA*	This study
RQC1485	RK5050, Δ*proA* + *proA*	This study
RQC1486	RK5046, Δ*proA* + *proA*	This study

### Mutant generation with in-frame deletion of *proA*

In the present study, mutants with an in-frame deletion of target genes were generated with the pK18mobsacB-based homolog recombination as described previously (Zhang et al., 2015). In brief, DNA fragments flanking both ends of target genes were conjugated with joint PCR and sub-cloned into pK18mobsacB. After validating the sequences, the plasmid was transferred into *R. solanacearum* strains by conjugation with *E. coli* S17-1. Mutants with an in-frame deletion of target genes were generated (listed in Table 1) and confirmed by colony PCR with respective primer pairs (Supplementary Table 1).

### Complementation analyses

In the present study, genetic complementation was performed with the Tn*7*-based site-specific chromosomal integration system as described previously that is a monocopy as the chromosome and is competent to fully restore phenotypes of mutants to those of parent strains (Zhang et al., 2011, 2021). In brief, the DNA fragment containing the coding sequence and native promoter (upstream region of about 500 bp empirically harboring the native promoter) was PCR amplified and cloned into pUC18-mini-Tn*7*T-Gm (Choi et al., 2005). After validating the sequence, the target DNA fragment was integrated into the chromosome of corresponding mutants at 25 bp of *glmS* downstream with the Tn*7*-based site-specific chromosomal integration system as monocopy (Zhang et al., 2021). Complementary strains were confirmed by colony PCR with a primer pair of glmsdown and Tn7R (Zhang et al., 2011).

### Bacterial growth assay

In the present study, a bacterial growth assay was performed both in medium (*in vitro*) and in tobacco leaves and tomato stems (*in planta*). Growth in the medium was assessed with optical densities at 600 nm (OD_600_). The *in planta* growth was represented in log_10_ cfu cm^–2^ in tobacco leaves and log_10_ cfu g^–1^ in tobacco stems, which were quantified by the dilution plating technique. L-proline and L-glutamate were purchased from Sango Biotech (Shanghai China) and supplemented into the minimal medium at a final concentration of 50 mM for the *in vitro* growth restoration assay. For the *in planta* growth assay in tobacco stems, tobacco plants were inoculated with the method of soil-soaking at a final cell density of 10^7^ cfu g^–1^ soil, and cell growth in tobacco stems was daily assessed. Each assay was performed with three biological replicates, including three replicates per trial for the *in vitro* growth assay and more than four biological replicates including six plants per trial for the *in planta* growth assay. Mean values of all experiments were averaged with SD, and the statistical significance was assessed using a *post-hoc* Dunnett test following ANOVA.

### β-Galactosidase assay

The β-galactosidase assay was performed to evaluate the expression levels of *lacZYA*-fused target genes both *in vitro* and *in planta* as described previously (Zhang et al., 2013). For the *in vitro* enzyme assay, bacterial strains were cultivated in a nutrient-rich medium or *hrp*-inducing minimal medium to an OD600 of approximately 0.1 and subjected to the enzyme assay, and the enzymatic activities were expressed in Miller Units (Miller, 1992). The *in planta* enzymatic activities were assessed with the Galacto-Light Plus kit and presented with RLU cell^–1^ (luminescence normalized by cell number) (Zhang et al., 2011). A bacterial suspension of 0.1 OD_600_ (10^8^ cfu ml^–1^) was infiltrated into the tobacco leaves, and bacterial cells in punched leaf disks were subjected to the *in planta* enzyme assay and cell counting by the dilution plating technique simultaneously. Each assay was performed with three biological replicates, including three replicates per trial for the *in vitro* enzyme assay and more than four biological replicates including six plants per trial for the *in planta* enzyme assay. Mean values of all experiments were averaged with SD, and the statistical significance was assessed using a *post-hoc* Dunnett test following ANOVA.

### Virulence assay

Virulence assay was carried out on wilt-susceptible tobacco plants (*Nicotiana tabacum* CV. Bright Yellow), which were grown for 3–4 weeks and subjected to virulence assay (Zhang et al., 2013). Briefly, tobacco plants were inoculated by methods of soil-soaking, which mimics natural invasion from rhizosphere soil into roots, and leaf infiltration, which enables direct invasion into the intercellular spaces of tobacco leaves (Zhang et al., 2013). Wilt symptoms of plants were assessed using 1–4 disease indexes, and the mean values of all experiments were averaged with SD. The virulence assay was also presented with survival curves, by which test plants were inspected daily with two levels of no wilting symptoms (disease index below 3) and completely wilted (disease index equal to or higher than 3) as previously described (Poueymiro et al., 2009). Each assay was carried out with at least four biological replicates including 12 plants per trial. The statistical significance was assessed using a *post-hoc* Dunnett test following ANOVA.

## Results

### ProA is essential for proline formation from glutamate in *Ralstonia solanacearum*

**FIGURE 1:**
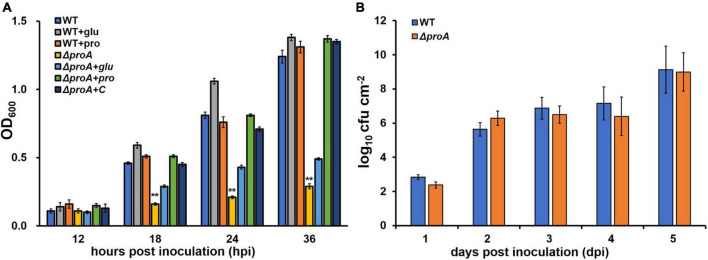
Involvement of ProA in the growth of *R. solanacearum* in **(A)** nutrient-limited sucrose medium (Hoagland medium with 2% sucrose) and **(B)** intercellular spaces of tobacco leaves. WT, the wild-type strain, RK5050 (OE1-1, *popA-lacZYA*); Δ*proA*, RQ5735 (RK5050, Δ*proA*); Δ*proA* + C, RQC1485 (complementary *proA* in RQ5735); + glu and + pro, supplemented glutamate at 100 mM or proline at 50 mM in the minimal medium, respectively. For the growth assay in medium, the cell suspension was washed two times with distilled water, adjusted to an OD_600_ of 1.0, and inoculated into the minimal medium with a proportion of 1%, and optical density at 600 nm (OD6_00_) was measured periodically. Each assay was repeated with three biological replicates, including three replicates per trial. For the growth assay in tobacco leaves, which was presented in log cfu cm^–2^, tobacco plants were inoculated with the method of leaf infiltration, and leaf disks (0.38 cm^2^) were punched and subjected to daily quantification of cell number by dilution plating. The growth assay was carried out till 5 dpi, when tobacco leaves became withered and dried. Each assay was repeated with more than four biological replicates, including six replicates per trial. Mean values of all experiments were averaged and presented with SD (error bars). Statistical significance between *proA* mutants and the wild-type RK5050, or that between treatments with supplementation or without supplementation, was assessed using a *post-hoc* Dunnett test following ANOVA. Significance level, ** indicates *P* < 0.01.

ProA catalyzes proline formation from glutamate in many bacteria (Hoffmann et al., 2017). We assessed whether ProA controls proline biosynthesis in *R. solanacearum*. The *proA* deletion mutant RQ5735 (RK5050, Δ*proA*), as well as the wild-type strain (RK5050, a *popA-lacZYA* reporter strain in *R. solanacearum* OE1-1), grew in the nutrient-rich broth medium (data not shown), but failed to grow in a minimal medium (Hoagland medium with 2% sucrose, *hrp*-inducing minimal medium) (Figure 1A). The genetic complementation assay was performed with the Tn*7*-based site-specific chromosomal integration system that is competent to fully restore the changed phenotypes of mutants (Choi et al., 2005; Zhang et al., 2021). Complementation of *proA* (RQC1485, Δ*proA* + C) fully restored the diminished growth of *proA* mutant in the minimal medium (Figure 1A), while supplementary proline (Δ*proA* + pro, 50 mM), but not glutamate (Δ*proA* + glu, 100 mM) fully restored the diminished growth of *proA* mutants in the minimal medium (Figure 1A), indicating that ProA controls proline formation from glutamate in *R. solanacearum*.

### The *proA* mutant grew normally inside tobacco leaves

Nutrients and oxygen inside the plants were believed to be relatively limited compared to those in a nutrient-rich broth medium, while *R. solanacearum* can utilize some secondary metabolites inside plants as carbon sources or substrates for the respiratory chain to fulfill colonization in host plants (Zuluaga et al., 2013; Lowe-Power et al., 2018). The *proA* mutants grew normally in a nutrient-rich broth medium, but failed to grow in the nutrient-limited minimal medium (Figure 1A). We assessed the proliferation of *proA* mutants in the tobacco leaves (intercellular spaces). For the growth assay in tobacco leaves, a bacterial suspension of 10^4^ cfu ml^–1^ was infiltrated into the intercellular spaces of tobacco leaves, and leaf disks were punched for daily quantification of cell densities with the dilution plating method (Zhang et al., 2015). The *proA* mutant RQ5735 (RK5050, Δ*proA*) proliferated similarly to the wild-type strain (RK5050) in tobacco leaves till 5 days post-infiltration (dpi) (Figure 1B). At this point, RK5050-infiltrated leaves became withered and dried. In consideration of the fact that *proA* mutants were proline auxotrophs (Figure 1A), proline in tobacco leaves might be enough for *proA* mutants to proliferate *in planta*.

### ProA is important for the type three secretion system expression both *in vitro* and *in planta*

**FIGURE 2:**
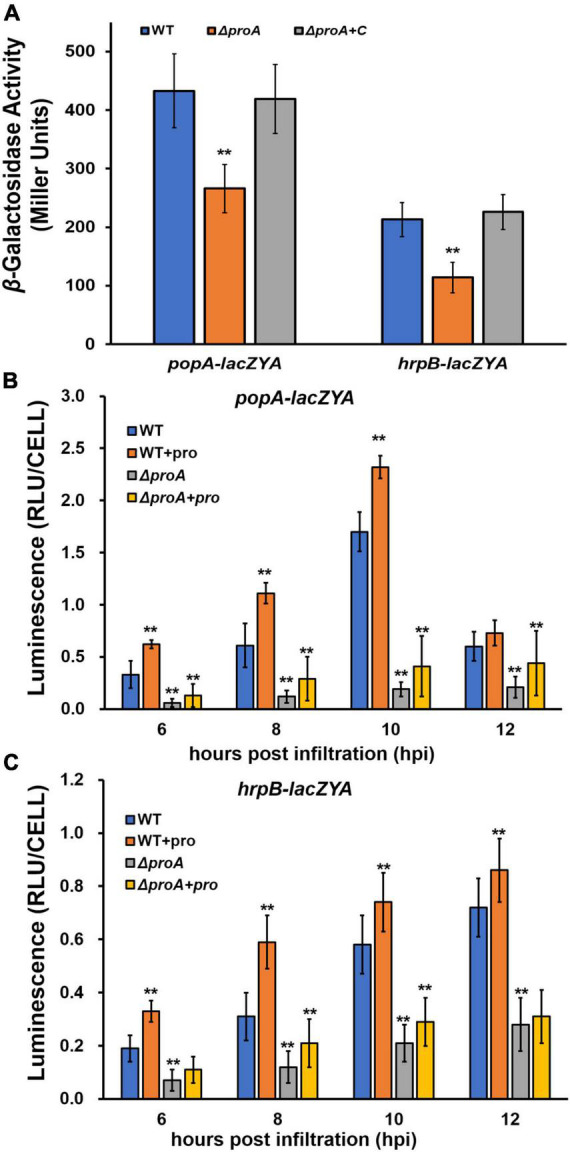
Involvement of ProA in the expression of *popA* (*popA-lacZYA*) and *hrpB* (*hrpB-lacZYA*). The wild type (WT) refers to reporter strains of RK5050 (OE1-1, *popA-lacZYA*) and RK5046 (*hrpB-lacZYA*); Δ*proA* refers to deletion of *proA* from RK5050 (OE1-1, *popA-lacZYA*) and RK5046 (*hrpB-lacZYA*). Δ*proA* + C refers to complementary *proA* in Δ*proA* mutant strains; + pro, supplemented proline into tobacco leaves. **(A)** Expression of *popA-lacZYA* and *hrpB-lacZYA* in *hrp*-inducing medium (the *in vitro* enzyme assay) with supplementary proline (50 mM); **(B,C)** expression of *popA-lacZYA* and *hrpB-lacZYA* in tobacco leaves (the *in planta* enzyme assay). For the *in vitro* enzyme assay, strains were grown in *hrp*-inducing medium with supplementary proline (50 mM) to an OD_600_ of about 0.1 and subjected to the enzyme assay, enzymatic activities of which were presented in Miller Units. Each assay was repeated with three biological replicates, including three replicates per trial. For the *in planta* enzyme assay, tobacco leaves were infiltrated with bacterial suspension of 0.1 OD_600_ or with supplementary proline (50 mM), and leaf disks were punched for the enzyme assay with the Galacto-Light Plus kit, enzymatic activities of which were presented with luminescence normalized by cells number (RLU cell^–1^). The *in planta* enzyme assay was carried out to 12 h post-infiltration (hpi), when infiltrated regions became dried. Luminescence was determined using the GloMax20 luminometer (Promega), and cell numbers in the punched leaf disks were quantified by dilution plating. Each assay was carried out with more than four biological replicates, including six replications per trial. Mean values of all experiments were averaged with SD, and statistical significance between the wild-type strains and mutants was assessed using a *post-hoc* Dunnett test following ANOVA. Significance level, ** indicates *P* < 0.01.

ProA was originally identified as one of the candidates that affected the expression of the T3SS in OE1-1 by transposon mutagenesis (Zhang et al., 2013). Expression of the T3SS in OE1-1 was monitored by a *popA-lacZYA* fusion, which exhibits similar expression profiles to the T3SS genes but did not alter the infection process of OE1-1 toward different host plants, including host invasion and colonization inside host plants (Yoshimochi et al., 2009b; Zhang et al., 2011, 2013). The expression of the T3SS is not activated in the nutrient-rich medium, but induced in a nutrient-limited medium (i.e., Hoagland medium with 2% sucrose, the so-called *hrp*-inducing medium) or by coming in contact with the host signals (Valls et al., 2006; Yoshimochi et al., 2009b). We first assessed whether ProA is required for the T3SS expression in an *hrp*-inducing medium. The *proA* mutants failed to grow in an *hrp*-inducing medium (minimal sucrose medium, Figure 1A), and proline was supplemented into this minimal sucrose medium at 50 mM to support the growth of *proA* mutants. Supplementary proline significantly enhanced *popA* expression of the wild-type strain (RK5050) in the *hrp*-inducing medium, which was about 1.5 fold higher than without supplementary proline (Figure 2A). Deletion of *proA* substantially impaired *popA* expression in the proline-supplemented minimal medium, in which *proA* mutants grew normally as the wild-type strain (Figure 2A). Complementation of *proA* (RQC1485, Δ*proA* + C) fully restored the impaired *popA* expression to levels of the wild-type strain (RK5050) in the proline-supplemented *hrp*-inducing medium (Figure 2A). HrpB plays a key regulatory role in the expression of the entire T3SS genes. We further evaluated whether ProA is required for *hrpB* expression in the *hrp*-inducing medium. Deletion of *proA* (RQ5935) substantially impaired *hrpB* expression in the proline-supplemented *hrp*-inducing minimal medium (Figure 2A). Complementary *proA* (RQC1486, Δ*proA* + C) fully restored the impaired *hrpB* expression to the levels of the parent strain RK5046 (OE1-1, *hrpB-lacZYA*) (Figure 2A), confirming that ProA is important for the expression of *hrpB* and the T3SS genes in the *hrp*-inducing medium.

The T3SS expression can be enhanced to much higher levels in the host plants by coming in contact with the host signals (Valls et al., 2006). We further assessed whether ProA is required for *popA* expression in the intercellular spaces of tobacco leaves. The *in planta* enzymatic activities were assessed with the Galacto-Light Plus kit and presented as RLU cell^–1^ (luminescence normalized by cell number) (Zhang et al., 2011). A bacterial suspension of 0.1 OD_600_ (10^8^ cfu ml^–1^) was infiltrated into the tobacco leaves, and bacterial cells in punched leaf disks were subjected to the *in planta* enzyme assay at 6–12 h post-inoculation (hpi). Enzymatic activities of the wild-type strain in tobacco leaves were about 0.4 RLU cell^–1^ at 6 hpi, increased to the maximum of about 1.6 RLU cell^–1^ at 10 hpi, and then decreased quickly to about 0.6 RLU cell^–1^ at 12 hpi (Figure 2B). Deletion of *proA* substantially impaired *popA* expression in tobacco leaves at 6–12 hpi, which got to the maximum of about 0.3 RLU cell^–1^ at 10–12 hpi (Figure 2B). Although proline in tobacco leaves might be enough for *proA* mutant to proliferate in tobacco leaves (Figure 1B), we also assessed whether the impaired *popA* expression of *proA* mutants in tobacco leaves was due to proline insufficiency. Extra proline (50 mM) was infiltrated into the tobacco leaves together with the bacterial suspension, and the *in planta* enzymatic activities were assessed. Consistent with the above results in the *hrp*-inducing medium, supplementary proline significantly enhanced *popA* expression of both the wild-type strain and *proA* mutants in tobacco leaves at 6–12 hpi, i.e., *popA* expression of the wild-type strain and *proA* mutants reached the maximum of about 2.3 and 0.5 RLU cell^–1^ at 10 and 12 hpi, respectively, which were significantly higher than those without proline supplementation (Figure 2B), indicating that proline is a novel inducer of the T3SS expression. The *popA* expression of *proA* mutants in proline-supplemented tobacco leaves was substantially lower than that of the wild-type strain in tobacco leaves with or without proline supplementation (Figure 2B).

The impact of ProA on *hrpB* expression was also carried out in tobacco leaves. Consistent with the above results on *popA* expression, supplementary proline significantly increased *hrpB* expression of both the wild-type strain and *proA* mutants in tobacco leaves at 6–12 hpi, while *hrpB* expression of *proA* mutants was significantly lower than that of the parent strain in tobacco leaves with or without proline supplementation (Figure 2C), confirming that ProA is important for the T3SS expression both *in vitro* and *in planta* and that ProA must have additional roles apart from proline biosynthesis in promoting the T3SS expression.

### Involvement of ProA in the type three secretion system expression is mediated through PrhG-HrpB pathway

**FIGURE 3:**
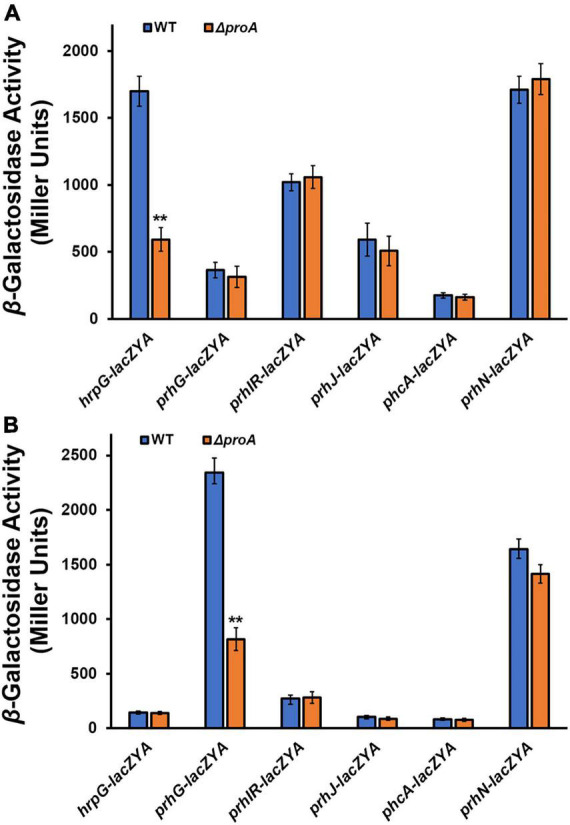
Involvement of ProA in the expression of T3SS-regulated genes in **(A)** rich medium and **(B)** in *hrp*-inducing minimal medium with supplementary proline (50 mM). Wild type (WT) refers to each reporter strain of RK5120 (*hrpG-lacZAY*), RK5212 (*prhG-lacZAY*), RK5619 (*prhN-lacZAY*), RQ6144 (*prhIR-laczYA*), RQ6074 (*prhJ-lacZYA*), and RK5043 (*phcA-lacZAY*). ΔproA refers to the deletion of *proA* from each reporter strain. Enzymatic activities were assessed with the *in vitro* enzyme assay and presented in Miller Units. Each assay was repeated with three biological replicates, including three replicates per trial. Mean values of all experiments were averaged with SD, and statistical significance between the wild-type strains and mutants was assessed using a *post-hoc* Dunnett test following ANOVA. Significance level, ** indicates *P* < 0.01.

HrpG and PrhG positively regulate *hrpB* expression in parallel ways, and some other regulators, i.e., PrhI/R, PrhJ, PhcA, and PrhN, regulate the expression of *hrpG* and *prhG*, respectively (Valls et al., 2006; Zhang et al., 2015; Hikichi et al., 2017). We further assessed whether deletion of *proA* alters the expression of these T3SS-regulating genes. Deletion of *proA* significantly impaired *prhG* expression in the proline-supplemented minimal medium (*hrp*-inducing medium), but not other genes (Figures 3A,B). Intriguingly, *hrpG* expression was significantly impaired with *proA* deletion but only in the nutrient-rich medium (Figure 3A). Four genes, *prhIR*, *prhJ*, *phcA*, and *prhN*, in *proA* mutants and parent strains were found to be expressed both in the nutrient-rich and proline-supplemented minimal media (Figures 3A,B). It is worth noting that both *proA* mutants and the wild-type strain grew in the nutrient-rich medium (data not shown), and the T3SS expression was not activated in the nutrient-rich medium. Involvement of ProA in the T3SS expression was mediated through PrhG-HrpB pathway under *hrp*-inducing conditions, but through some novel pathways to PrhG.

### ProA is essential for pathogenicity of *Ralstonia solanacearum* in host plants

**FIGURE 4:**
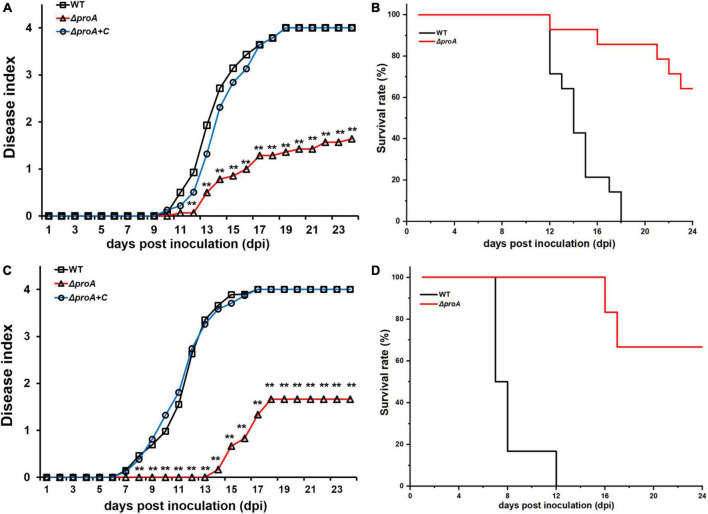
Virulence test of *proA* mutants in tobacco plants with the inoculation method of **(A,B)** leaf infiltration, which enables direct invasion into intercellular spaces of leaves, and **(C,D)** soil-soaking inoculation, which mimics natural invasion through roots. WT, the wild-type strain, RK5050 (OE1-1, *popA-lacZYA*); Δ*proA*, RQ5735 (RK5050, Δ*proA*). Δ*proA* + C, RQC1485 (complementary *proA* in RQ5735). For the leaf infiltration, about 50 μl of the bacterial suspension of 10^8^ cfu ml^–1^ was infiltrated into the tobacco leaves with a blunt-end syringe. For the soil-soaking inoculation, a bacterial suspension was poured into a pot at a final cell density of 10^7^ cfu g^–1^ of soil. Panels **(A,C)** refer to the virulence test with the disease index. Wilt symptoms were inspected daily and scored on a disease index scale from 0 to 4 (0, no wilting; 1, 1–25% wilting; 2, 26–50% wilting; 3, 51–75% wilting; 4, 76–100% wilted or dead). Panels **(B,D)** refer to surviving curves corresponding to the virulence test with the disease index of panels **(A)** and **(B)**, respectively. Mean values of all experiments were averaged with SD. But, the SD was not presented in figures for virulence assay due to the consideration of esthetic appearance. Statistical significance was assessed using a *post-hoc* Dunnett test following ANOVA. Significance level, ** indicates *P* < 0.01.

Deletion of *proA* substantially impaired the expression of the T3SS, which is essential for the pathogenicity of *R. solanacearum* (Genin and Denny, 2012). We evaluated whether ProA contributed to the pathogenicity of *R. solanacearum*. Tobacco plants were subjected to the virulence assay with inoculation methods of both soil-soaking, which mimics natural host invasion through roots, and leaf infiltration, which enables direct invasion into the intercellular spaces of leaves (Zhang et al., 2011). The wild-type strain (RK5050) wilted all test tobacco plants at about 18 dpi with the inoculation method of leaf infiltration (Figures 4A,B), and at about 12 dpi with the method of soil-soaking (Figures 4C,D). The *proA* mutant (RQ5735) wilted approximately 30% of the tobacco plants till 24 dpi, and complementation of *proA* (Δ*proA* + C) fully restored the impaired virulence of *proA* mutants to that of the wild-type strain (Figure 3), confirming that ProA is important for *R. solanacearum* to wilt host tobacco plants.

### ProA is important for *Ralstonia solanacearum* to invade and colonize host xylem vessels

**FIGURE 5:**
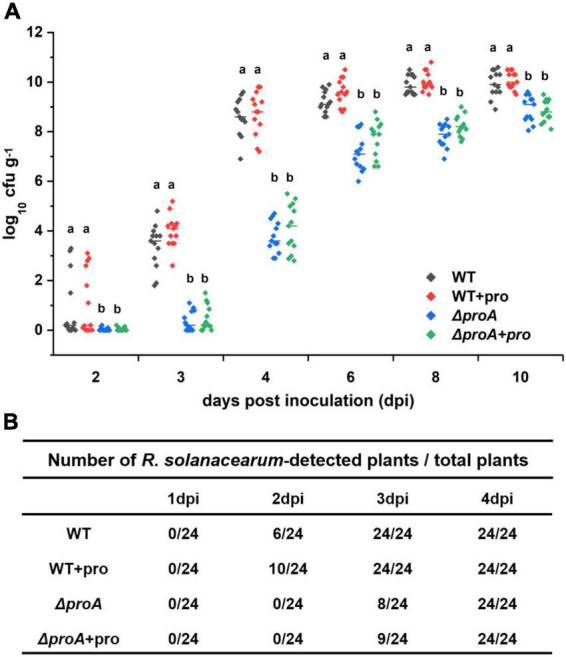
Involvement of ProA in the invasion and colonization of *R. solanacearum* in tobacco xylem vessels. WT, the wild-type strain, RK5050 (OE1-1, *popA-lacZYA*); Δ*proA*, RQ5735 (RK5050, Δ*proA*); + pro, supplemented proline into pot soil. **(A)** Daily growth of *R. solanacearum* strains in tobacco xylem vessels; **(B)** percentage of plants colonized by *R. solanacearum* strains. For the proline supplementation, tobacco plants were irrigated with one-quarter of diluted Hoagland medium with supplementary proline at 10 mM for five times and subjected to bacterial inoculation. For the growth assay in tobacco xylem vessels, tobacco plants were inoculated with the inoculation method of soil-soaking, which mimics natural invasion through roots, and stem species were cut, weighted, and subjected to quantification of cell number by the dilution plating. Bacterial growth in xylem vessels was presented in log cfu g^–1^. Each assay was repeated with more than four biological replicates, including six plants per trial. Mean values of all experiments were averaged with SD, and statistical significance between the wild-type strains and mutants was assessed using a *post–hoc* Dunnett test following ANOVA.

As a soil-borne vascular plant pathogenic bacterium, *R. solanacearum* generally invades host xylem vessels through natural root openings or root wounds and subsequently invades xylem vessels, in which *R. solanacearum* proliferates extensively and produces copious amounts of exopolysaccharide (EPS) slime to block sap flows (Vasse et al., 1995; Genin, 2010; Milling et al., 2011). The *proA* mutants were proline auxotrophs that failed to grow in the minimal sucrose medium (Figure 1A), while they could eventually wilt part of tobacco plants even with the soil-soaking inoculation method, which mimics natural root invasion. We evaluated whether the deletion of *proA* impaired the abilities to invade and colonize tobacco xylem vessels. Tobacco plants were inoculated with the method of soil-soaking, and bacterial densities in xylem vessels were determined till 10 dpi. At this point, most of the RK5050-inoculated tobacco plants exhibited obvious wilting symptoms (Figure 4C). The wild-type strain (RK5050) could not be detected in tobacco xylem vessels at 1 dpi, but could be detected in about one-quarter of test xylem vessels at 2 dpi and could be detected in all test xylem vessels at 3 dpi (Figure 5). *R. solanacearum* could successfully invade tobacco xylem vessels at about 2–3 dpi with the inoculation method of soil-soaking. The *proA* mutants could not be detected in tobacco xylem vessels at 2 dpi, but could be detected in about one-third of test xylem vessels at 3 dpi and could be detected in all test xylem vessels at 4 dpi (Figure 5). The *proA* mutants could successfully invade tobacco xylem vessels at about 3–4 dpi with the inoculation method of soil-soaking, which was about 1-day delay compared to that of the wild-type strain. The wild-type strain proliferated to approximately 10^4^ cfu g^–1^ at 3 dpi, reached the maximum of approximately 10^10^ cfu g^–1^ at 8 dpi and maintained this maximum density till 10 dpi (Figure 5), while *proA* mutants proliferated to the maximum density of approximately 10^9^ cfu g^–1^ at 10 dpi (Figure 5), which was about one order of magnitude less and more slowly than that of the wild-type strain to proliferate to the maximum density in tobacco xylem vessels. Deletion of *proA* significantly impaired the ability of *R. solanacearum* to invade and colonize host xylem vessels.

We further assessed whether the impaired abilities of *proA* mutants to invade and colonize tobacco xylem vessels were due to proline insufficiency in rhizosphere soil. Tobacco plants were irrigated with one-quarter of diluted Hoagland medium with supplementary proline at 10 mM for five times and subjected to bacterial inoculation. Supplementary proline slightly enhanced the invasion rate of the wild-type strain into the tobacco xylem vessels, i.e., the wild-type strain (RK5050) could be detected in about 42% of test tobacco xylem vessels at 2 dpi, but no significant impact on proliferation inside the xylem vessels at 3–10 dpi was observed (Figure 5). On the other hand, supplementary proline did not alter the ability of *proA* mutants to invade and colonize tobacco xylem vessels at 2–10 dpi (Figure 5), indicating that impaired abilities of *proA* mutants to invade and colonize tobacco plants is independent of proline insufficiency in the rhizosphere soil.

## Discussion

We here provided multiple lines of evidence to demonstrate that ProA controls proline biosynthesis in *R. solanacearum*, while it displays additional roles apart from proline biosynthesis in promoting the expression of the T3SS and host invasion of *R. solanacearum*. The *proA* mutants were proline auxotrophs that failed to grow in the minimal medium. Supplementary proline, but not glutamate, fully restored the diminished growth of *proA* mutants in the minimal medium, confirming that ProA catalyzes proline synthesis from glutamate. It was consistent with the pioneering reports that ProA catalyzes the synthesis of l-glutamic acid 5-semialdehyde from gamma-glutamyl phosphate, which is the second reaction of proline formation from glutamate in many bacteria (Krishna et al., 1979; Hoffmann et al., 2017). Nutrients inside host plants were known to be relatively limited compared to that in nutrient-rich broth medium, but relatively more abundant than that in minimal sucrose medium, since plants synthesized abundant secondary metabolites and *R. solanacearum* can utilize some of the plant-synthesized secondary metabolites, such as sucrose and putrescine, as the sole carbon source to fulfill the *in planta* proliferation (Lowe-Power et al., 2017, 2018). Fluids collected from intercellular spaces of tobacco leaves greatly enhance the T3SS expression to higher levels than that in an *hrp*-inducing medium, indicating that nutrient conditions in intercellular spaces of tobacco leaves were relatively limited (Mori et al., 2016). The *proA* mutants, proline auxotroph mutants, as well as the wild-type strain grew in the intercellular spaces of tobacco leaves, indicating that proline in tobacco leaves might be sufficient for *proA* mutants to proliferate inside tobacco leaves. We previously reported that some *R. solanacearum* auxotrophic mutants, i.e., cysteine auxotrophic mutants and aromatic amino acids auxotrophic mutants, failed to grow in the minimal sucrose medium, but grew slowly inside the host tomato and tobacco plants (Zhang et al., 2019a,b; Chen et al., 2022), indicating that nutrients inside host plants were relatively more abundant than those in the minimal sucrose medium, and thus *R. solanacearum* auxotrophic mutants could grow inside host plants to some extent.

The T3SS plays an essential role in the infection process of *R. solanacearum* toward different host plants, expression of which was activated in an *hrp*-inducing medium and enhanced to much higher levels inside the host plants or when it comes in contact with plant signals. Interestingly, proline was demonstrated as a novel T3SS inducer that significantly enhanced the expression levels of *popA* (representative gene of the T3SS) and *hrpB* (master regulator of the T3SS) both *in vitro* (*hrp*-inducing medium) and *in planta* (intercellular spaces of tobacco leaves). Although supplementary proline significantly enhanced the expression levels of *popA* and *hrpB* in *proA* mutants both *in vitro* and *in planta*, it was substantially lower than those of the parent strains, i.e., about one-third to one-quarter levels of the wild-type strain in proline-supplemented tobacco leaves (Figure 2B), confirming that ProA has a novel impact on the T3SS expression. Note that *proA* mutants grew well in the proline-supplemented minimal medium and in tobacco leaves. The impact of ProA on the T3SS expression is independent of growth deficiency under nutrient-limited conditions. Similar results were also reported in some *R. solanacearum* auxotrophic mutants, i.e., the T3SS expression in cysteine auxotrophic mutants and aromatic amino acids auxotrophic mutants, which failed to grow in the minimal sucrose medium but grew slowly inside host plants and exhibited significantly impaired T3SS expression (Zhang et al., 2019b; Chen et al., 2022). Thus, ProA might play additional roles apart from proline biosynthesis in promoting the T3SS expression in *R. solanacearum*.

The *hrpB* expression was substantially impaired in *proA* mutants, which was consistent with the master regulatory role of HrpB that directly controls the expression of the entire T3SS and T3Es genes (Mukaihara et al., 2004; Valls et al., 2006). Two close paralogs, HrpG and PrhG, positively regulate *hrpB* expression in parallel ways (Plener et al., 2010; Zhang et al., 2013). Deletion of *proA* substantially impaired *prhG* expression in the minimal medium, while it did not alter the expression of known regulatory genes, *prhIR*, *prhJ*, *prhN*, and *phcA*, which regulate the expression of the T3SS genes indirectly. Note that the T3SS expression is not initiated in the rich medium, but activated in the minimal medium that might mimic the conditions of plant apoplastic fluids. Involvement of ProA in the T3SS expression is mediated through the pathway of PrhG to HrpB, at least for the *in vitro* T3SS expression. Intriguingly, *proA* deletion substantially impaired *hrpG* expression in the nutrient-rich broth medium. Both HrpG and PrhG are essential for the T3SS expression, while PrhG contributes partially to the *in planta* T3SS expression, host colonization, and pathogenicity, which might respond to metabolic signals or some mimic host signals, and HrpG is essential for the *in planta* T3SS expression, host invasion, colonization, and pathogenicity (Plener et al., 2010; Zhang et al., 2013; Pedro-Jove et al., 2021). Note that the expression levels of *hrpG* in the rich medium are much higher than those in the minimal medium, while the T3SS expression is not activated (Yoshimochi et al., 2009b). Posttranslational modification of HrpG and PrhG is essential for their activation of the expression of *hrpB* and the T3SS. Deletion of *proA* might impair not only the transcriptional activity inside the host plants but also the posttranslational modification of HrpG and PrhG. It is not a special case, and we previously reported that CysB, a regulator of cysteine synthesis, positively regulates the expression of the T3SS genes both *in vitro* and *in planta* through the pathway PrhG to HrpB, while the impairment of the expression of the T3SS genes in *cysB* mutants was independent of growth deficiency under nutrient-limited conditions (Chen et al., 2022). Moreover, the T3SS expression is positively regulated by a PadR regulator PrhP through the key regulator HrpB but via some novel pathway to HrpB (Zhang et al., 2019b). All these results support the speculation that some novel pathways should be integrated for the posttranslational modification of HrpG and PrhG, and in turn regulate the expression of the T3SS in *R. solanacearum*. It remains to be further elucidated whether and how ProA is involved in the posttranslational modification of HrpG and PrhG, and in turn controls the expression of the T3SS genes in *R. solanacearum*.

As a soil-borne vascular plant pathogenic bacterium, the ability to invade and colonize host xylem vessels is critical for the pathogenicity of *R. solanacearum* (Vasse et al., 1995; Genin, 2010). The *proA* mutants could grow inside tobacco plants, while its proliferation in tobacco xylem vessels was significantly impaired, which was about one order of magnitude less than the wild-type strain to proliferate to the maximum cell densities in xylem vessels and was about 1–2 days to proliferate to the maximum cell densities in xylem vessels. Moreover, the ability of *proA* mutants to naturally invade tobacco xylem vessels was significantly impaired, which was about 1-day delay for *proA* mutants to invade tobacco xylem vessels from roots compared with the wild-type strain. Natural invasion into tobacco roots and proliferation of *proA* mutants in tobacco xylem are possibly the main clues for its substantially impaired virulence in tobacco plants. The T3SS is essential for *R. solanacearum* to successfully invade host plants, particularly at the early stage of invading host cells (Cunnac et al., 2004; Angot et al., 2006; Jones and Dangl, 2006). Supplementary proline in pot soil did not restore impaired abilities of *proA* mutants to invade and colonize xylem vessels, indicating that impaired abilities in *proA* mutants might be due to substantially impaired T3SS expression, but not due to proline insufficiency in the rhizosphere soil or inside the host plants.

All taken together, these results provide novel insights into the understanding of the biological functions of ProA, an enzyme required for the biosynthesis of proline, and its involvement in the T3SS in *R. solanacearum*. ProA displays novel roles apart from proline biosynthesis and functions in promoting the expression of the T3SS genes and host invasion in *R. solanacearum*.

## Data availability statement

The original contributions presented in this study are included in the article/Supplementary material, further inquiries can be directed to the corresponding author/s.

## Author contributions

YZ and JL conceived and designed the experiments. YG, RW, YZ, NC, and LH performed the experiments. JL and YOZ analyzed and discussed the results. JL wrote the manuscript. YOZ revised the manuscript. All authors contributed to the article and approved the submitted version.
