# Supplementary material for: Functional characterization of a gamma-glutamyl phosphate reductase ProA in proline biosynthesis and promoting expression of type three secretion system in Ralstonia solanacearum
Source: Front Microbiol. 2022 Aug 29;13:945831. doi: 10.3389/fmicb.2022.945831 (PMC9465252; doi:10.3389/fmicb.2022.945831)
Supplement: Supplementary file 1 [file Table_1.DOCX]

**Table S1. Primers used in this study**

| primer | sequence | Reference | |
| --- | --- | --- | --- |
| proAA1B | ATGGATCCGCGCTACGACGTCTTCAAG | | This study |
| proAB1C | GCGCAGCGTGCGCGACGGTAGATGATCGCGACCGGCGGGA | | This study |
| proAA2C | TCCCGCCGGTCGCGATCATCTACCGTCGCGCACGCTGCGC | | This study |
| proAB2H | CGAAGCTTCGTTGAACCAGCGGTAGA | | This study |
| glmsdown | GCGCTCAAGCTCAAGGAGATC | Zhang *et al*., 2011 | |
| Tn7R | CACAGCATAACTGGACTGATTTC | Choi *et al*., 2005 | |
